# MS4a4B, a CD20 Homologue in T Cells, Inhibits T Cell Propagation by Modulation of Cell Cycle

**DOI:** 10.1371/journal.pone.0013780

**Published:** 2010-11-01

**Authors:** Hui Xu, Yaping Yan, Mark S. Williams, Gregory B. Carey, Jingxian Yang, Hongmei Li, Guang-Xian Zhang, Abdolmohamad Rostami

**Affiliations:** 1 Department of Neurology, Thomas Jefferson University, Philadelphia, Pennsylvania, United States of America; 2 Department of Microbiology and Immunology, University of Maryland School of Medicine, Baltimore, Maryland, United States of America; New York University, United States of America

## Abstract

MS4a4B, a CD20 homologue in T cells, is a novel member of the MS4A gene family in mice. The MS4A family includes CD20, FcεRIβ, HTm4 and at least 26 novel members that are characterized by their structural features: with four membrane-spanning domains, two extracellular domains and two cytoplasmic regions. CD20, FcεRIβ and HTm4 have been found to function in B cells, mast cells and hematopoietic cells respectively. However, little is known about the function of MS4a4B in T cell regulation. We demonstrate here that MS4a4B negatively regulates mouse T cell proliferation. MS4a4B is highly expressed in primary T cells, natural killer cells (NK) and some T cell lines. But its expression in all malignant T cells, including thymoma and T hybridoma tested, was silenced. Interestingly, its expression was regulated during T cell activation. Viral vector-driven overexpression of MS4a4B in primary T cells and EL4 thymoma cells reduced cell proliferation. In contrast, knockdown of MS4a4B accelerated T cell proliferation. Cell cycle analysis showed that MS4a4B regulated T cell proliferation by inhibiting entry of the cells into S-G2/M phase. MS4a4B-mediated inhibition of cell cycle was correlated with upregulation of Cdk inhibitory proteins and decreased levels of Cdk2 activity, subsequently leading to inhibition of cell cycle progression. Our data indicate that MS4a4B negatively regulates T cell proliferation. MS4a4B, therefore, may serve as a modulator in the negative-feedback regulatory loop of activated T cells

## Introduction

MS4a4B is a novel member of the MS4A gene family (membrane-spanning 4-domain family, subfamily A, MS4As) which is characterized by their structural features, with four membrane-spanning domains, two extracellular domains and two cytoplasmic regions [1]. The MS4A family includes CD20, FcεRIβ, HTm4 and at least 26 novel members [2], [3]. Chromosome mapping shows that the genes for human CD20, FcεRIβ, HTm4 and 12 recently identified MS4A members are located in chromosome 11q12-q13 [4], [5], which is associated with increased susceptibility to allergy and atopic asthma. The genes for mouse CD20 and FcεRIβ are located in chromosome 19 [6], [7]. The gene clustering and the chromosomal localization of the MS4A family may suggest their immunological relevance. So far, our knowledge of the MS4A family is derived mainly from studies on CD20, HTm4 and FcεRIβ. CD20 is a nonglycosylated, plasma-membrane associated protein in B cells [7], [8], which disappears when B cells differentiate into plasma cells [9], [10]. Early studies show that CD20 functions in B cells as a Ca^2+^ channel or Ca^2+^ channel regulator [11]. However an increasing body of data suggests that CD20 is not only involved in calcium signaling but also more extensively associated with B cell activation, differentiation and apoptosis [12], [13]. Moreover, CD20 has been used as the target of anti-CD20 treatment for B cell lymphoma and autoimmune diseases, which to date has been considered as the most successful antibody-based therapeutics [14]. In comparison with CD20, HTm4 is predominantly expressed on nuclear membrane in hematopoietic lineages and is functionally associated with differentiation of hematopoietic cells [15]. Unlike CD20 and HTm4, FcεRIβ, as a part of the receptor complex for IgE Fc fragment, contains an immunoreceptor tyrosine activation motif (ITAM) in its C-terminal cytoplasmic domain that directly contributes to IgE binding-mediated cell signalling [16], [17], [18]. The functions of other members remain largely unclear. Since we cloned MS4a4B from the thymus of C57BL/6 mice, data from our studies and others have shown that MS4a4B is highly expressed in T cells and is closely related to the regulation of CD4^+^ T cell-mediated immune responses [1], [19], [20], suggesting its importance in adaptive immunity.

Involvement of MS4A proteins in cell proliferation and cell cycle regulation has been suggested by studies with CD20 and HTm4 [13], [15]. It has been shown that Epstein-Barr viral vector-driven expression of CD20 in fibroblasts accelerates G1 progression in a Ca^2+^-dependent manner [21]. However surface cross-linking of CD20 with different anti-CD20 monoclonal antibodies generates the opposite results: cross-linking of CD20 with anti-B1a antibody inhibits B cell progression into the S/G2+M stages of the cell cycle [7], [22] and drives B cells to undergo apoptosis [4], [5] but binding of anti-CD20 monoclonal antibody 1F5 to CD20 can activate B cells and initiate cell cycle transition from G0 to G1 phase [23]. In contrast, overexpression of HTm4 in U937 cells inhibits the G1-S transition of cell cycle through interaction with cyclin-dependent kinase-associated (CDK-associated) phosphatase-CDK2 (KAP-CDK2) complexes [15], [24]. It remains unclear whether other members of the MS4A family, including MS4a4B, play roles in cell cycle and cell proliferation. Given that CD20 is critical for cell proliferation and cell cycle regulation in B cells, and serves as a target for anti-CD20-based immune therapeutics of B cell-related diseases [25] we are encouraged to dissect the biological function of MS4a4B in T cells.

In this report, we demonstrate that although MS4a4B is expressed at high levels in mature T cells, its expression is silenced in malignant T cells. We analyzed the impact of MS4a4B on T cell proliferation by manipulating MS4a4B expression with MS4a4B-expressing and –silencing approaches. We found that MS4a4B negatively regulates T cell proliferation by interfering with cell cycle progression from G0/G1 phase into S-G2/M phases through inhibition of the Cdk2-Rb pathway, and that silence of MS4a4B in thymoma is, at least partially, responsible for the uncontrollable propagation of tumor T cells.

## Results

### MS4a4B is expressed in mature T cells but not in malignant T cells

**Figure 1:**
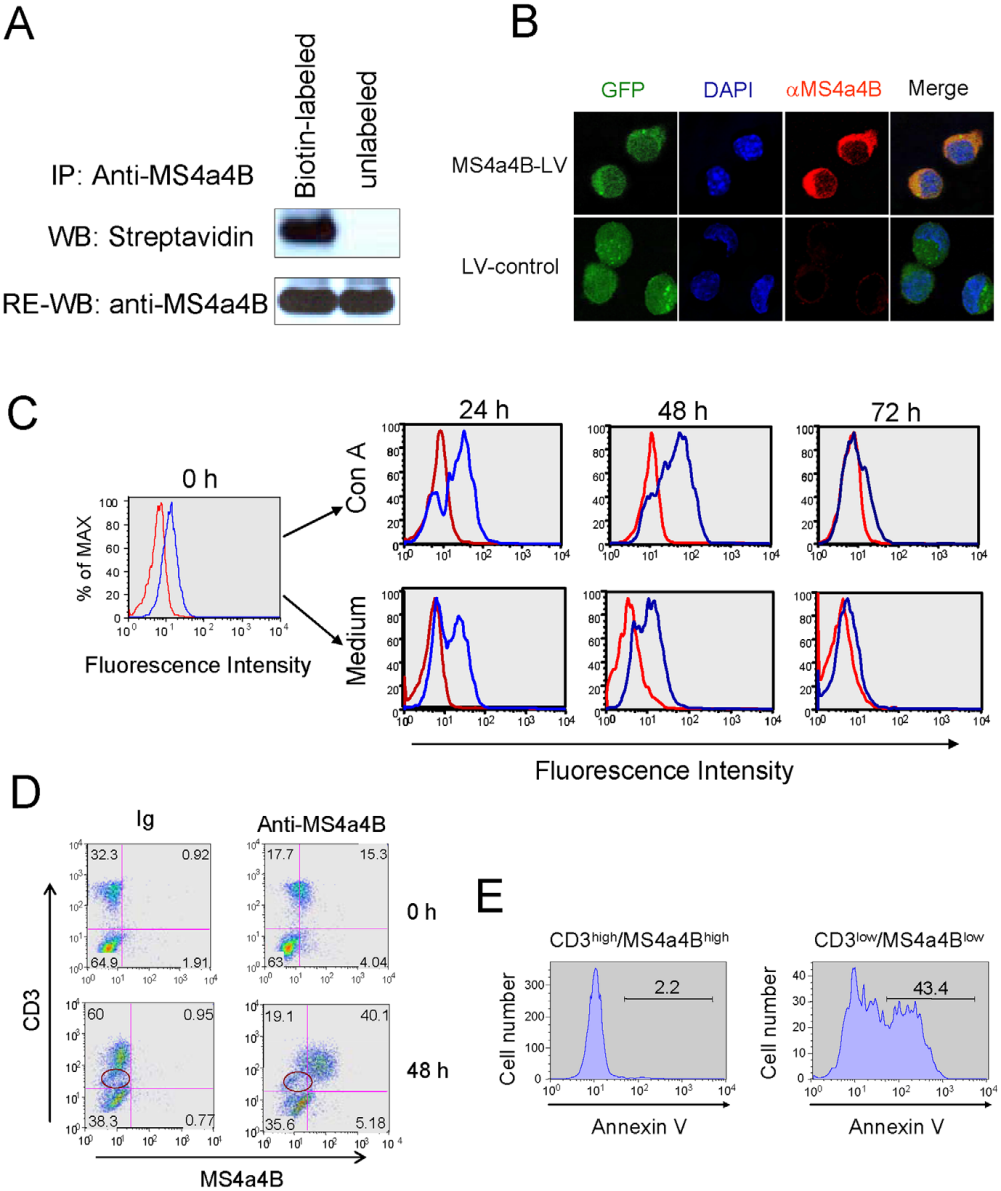
MS4a4B is expressed on cell surface and is potentially associated with activation of T cells. **A,** To determine whether MS4a4B is expressed on cell surface, mouse spleen cells were surface-labeled with EZ-Link Sulfo-NHS-SS-Biotin according to the manufacturer's protocol (Pierce). Unlabeled spleen cells were used as control. Cells were lysed by lysis buffer containing 1% NP-40. Cell lysate was immunoprecipitated by anti-MS4a4B-coupled protein A-beads and was separated on 12% SDS-PAGE, followed by blotting with streptavidin-HRP. MS4a4B was confirmed by re-blotting with anti-MS4a4B antibody. **B,** EL4 cells, infected with either MS4a4B-expressing lentivirus (LV) vector or mock LV vector, were stained with rabbit anti-MS4a4B antibody followed by labeling with anti-rabbit-IgG-Cy3 conjugate. Expression and localization of MS4a4B were observed by confocal microscopy. Magnification, ×40. **C,** Spleen cells were cultured for 24, 48 and 72 hrs in the presence or absence of Con A (5 µg/ml). Spleen cells before culture were used as control (0 hr). Cells were first stained with anti-CD3-PE, and then intracellular stained with biotinylated anti-MS4a4B antibody (blue line) or biotinylated Ig control (red line), followed by labeling with streptavidin-Red 670. For flow cytometric analysis, cells were first gated on CD3, and then were analyzed for MS4a4B expression. **D,** Spleen cells pre- or 48 hr post Con A stimulation were co-immunostained with anti-CD3 and anti-MS4a4B antibody. Cells were analyzed by flow cytometry. Note the CD3^low^/MS4a4B^low^ population. **E,** Spleen cells were stimulated with Con A for 48 hr and were co-stained with Annexin V, anti-CD3 and anti-MS4a4B antibodies. CD3^low^/MS4a4B^low^ and CD3^high^/MS4a4B^high^ populations were gated for assessment of apoptosis indicated by Annexin V binding.

To define the expression of MS4a4B, we detected MS4a4B protein in mouse cells, including normal T cells, non-T cells, and malignant T cells, by using anti-MS4a4B antibodies and flow cytometric analysis. Consistent with our previous findings [1], MS4a4B was strongly expressed in naïve T cells but was not expressed in B cells. In addition to T cells, we also examined expression of MS4a4B in other cells. The results showed that MS4a4B was also expressed at high levels in NK cells (marked by NK1.1) and moderately expressed in macrophages (marked by Mac1) (supplementary Fig. S1A). In bone marrow cells, only the Mac1^+^ population expressed low levels of MS4a4B, suggesting MS4a4B expression in early hematopoietic progenitors (supplementary Fig. S1A). Surprisingly, all malignant T cells, including thymoma and T hybridoma cell lines that we have examined so far, lose expression of MS4a4B (Table 1 and supplementary Fig. S2). Lack of MS4a4B protein in thymoma cells led us to postulate that MS4a4B may be required for appropriate functioning of mature T cells and the absence of this protein may be, at least in part, responsible for the uncontrollable growth of thymoma. Western blotting with surface-biotin labeling of primary T cells and histological studies with confocal microscopy of MS4a4B-expressing retrovirus-infected EL4 thymoma cells showed that MS4a4B was indeed expressed on cell surface (Fig. 1A and B), suggesting that MS4a4B may potentially interact with other cell membrane proteins in T cell regulation. Interestingly, expression of MS4a4B is regulated not only during thymocyte development [1] but also during primary T cell activation (Fig. 1C). Although MS4a4B was constitutively expressed in primary naïve T cells, levels of its expression were markedly increased upon stimulation by mitogen concanavalin A (Con A). However, expression of MS4a4B was gradiently decreased by 72 hour of activation. It is of note that the down-regulation of MS4a4B was closely associated with reduction of surface CD3 expression in T cells (Fig. 1D), which led us to speculate whether this CD3^low^/MS4a4B^low^ population might represent the cells undergoing apoptosis. We further examined apoptosis of this population by Annexin V assay. The CD3^low^/MS4a4B^low^ population showed markedly high levels of apoptotic cells (Fig. 1E). Thus, temporal expression of MS4a4B in activated T cells and silence of the MS4a4B gene in malignant T cells led us to hypothesize that MS4a4B may play a regulatory role in propagation of T cells.

**Table 1:** Expression of MS4a4B in mouse T cells and non-T cells.

Primary T cell		T cell line		Malignant T cell^b^		Non-T cell	
Cell	MS4a4B	Cell	MS4a4B	Cell	MS4a4B	Cell	MS4a4B
CD4^+^	+++^a^	T32	+++	EL4	−	B cell	−
CD8^+^	+++	AE7	+++	TIB47	−	NK	+++
NKT	+++	5CC7	+++	CRL1778	−	Bone marrow	+
		HT2	−	2993	−	Macrophage	+
		CTLL	−	T-180	−		
				58α	−		
				α28	−		

a MS4a4B expression was determined by flow cytometric analysis with anti-MS4a4B intracellular staining. Negative is indicated as “−”; positive is indicated by expression levels based on fluorescent intensity as low (“+”), middle (“++”) and high (“+++”).

b EL4, TIB47, CRL1778, 2993 and T180 are thymoma cell lines; 58α and α28 cells are T hybridoma.

### Inhibitory role of MS4a4B in T cell proliferation

**Figure 2:**
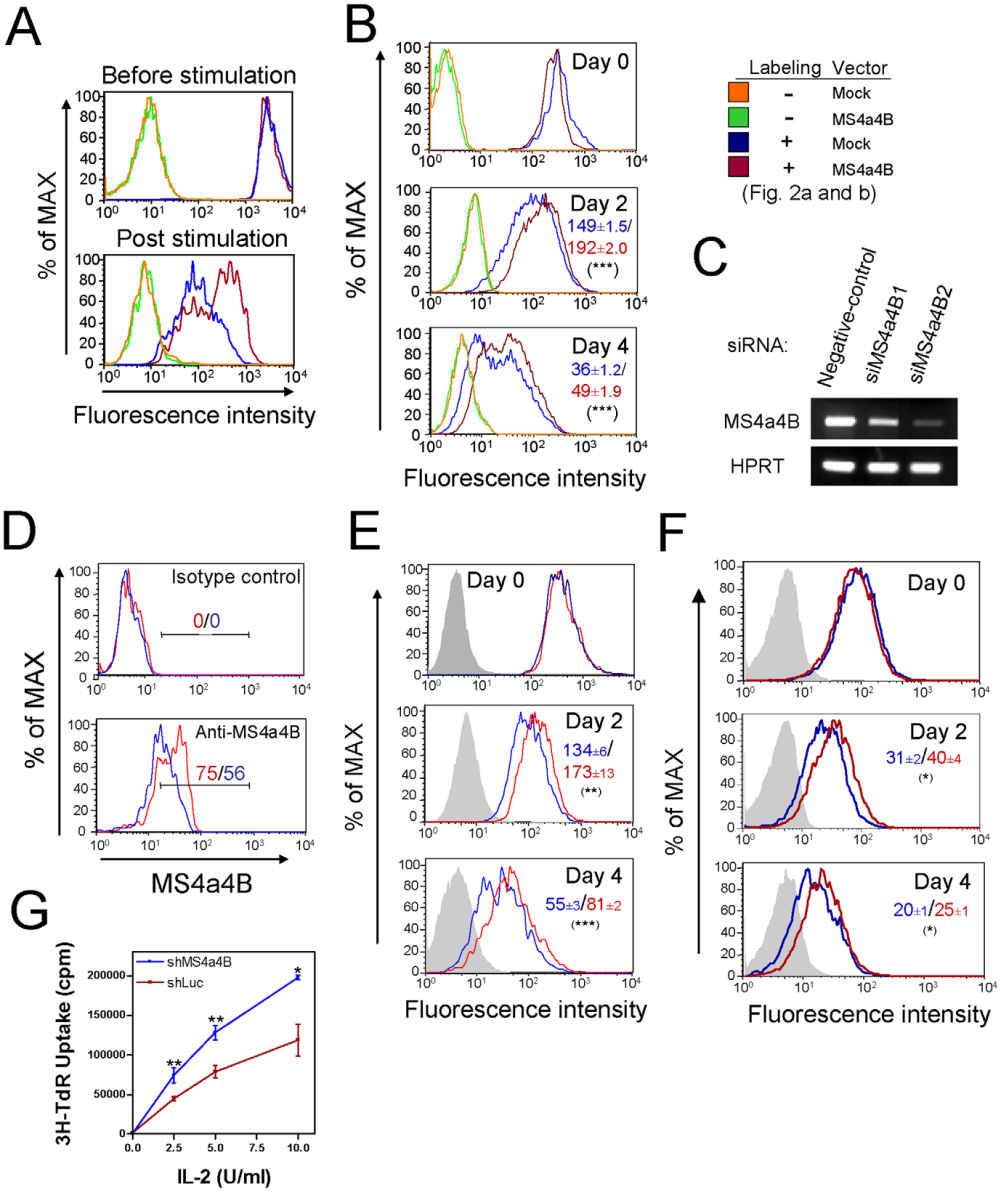
MS4a4B negatively regulates T cell proliferation. **A,** Retrovirus-driven over-expression of MS4a4B inhibits proliferation of primary T cells. Primary CD4^+^ T cells isolated from spleen with anti-CD4 magnetic beads were stimulated by anti-CD3/ anti-CD28 antibodies and then were infected with MS4a4B-retroviral vector or mock vector. After infection, cells were labeled with PKH-26 Red Fluorescent linker kit. Proliferation of the tested cells was analyzed by flow cytometry (FL2). Data shown are representative of three repeat experiments. **B,** Forced expression of MS4a4B by LV-vector reduced proliferation of EL4 cells. Purified EL4 cells with stable lentiviral infection were labeled with PKH-26 Red and then cultured in complete RPMI 1640 medium. Cells were collected on the days indicated. Cell proliferation was assessed by flow cytometry. Data shown are representative of three repeat experiments. The numbers in histograms are mean fluorescence intensity (MFI) ± SD. ***, p<0.001. **C,** Knockdown of MS4a4B by siRNA. T32 cells were transfected with either FAM-labeled negative control siRNA or FAM-labeled MS4a4B-specific siRNAs (siMS4a4B1 and siMS4a4B2). Cells were collected on day 3 after transfection. Transcription of MS4a4B mRNA was analyzed by RT-PCR. **D,** Cell samples collected from culture on day 4 were subjected to MS4a4B protein detection by flow cytometry with anti-MS4a4B antibody. Data are presented as fluorescence intensity of FL-2 in FAM-positive population. Blue line: siMS4a4B2-transfected cells; red line: control siRNA-transfected cells. **E** and **F,** Knockdown of MS4a4B expression accelerated proliferation of T32 cells (E) and primary T cells (F). T32 cells or anti-CD3/anti-CD28-stimulated CD4^+^ primary T cells were labeled with PKH-26 Red and were transfected with FAM-labeled negative control siRNA or FAM-labeled siMS4a4B2. The labeled cells were stimulated with complete medium containing 20 U/ml IL-2. Cells were collected from culture at the time indicated for determination of proliferation rate by flow cytometric analysis. Data are presented as representative of three repeat experiments. Tinted grey peak: unlabeled cells; blue line: siMS4a4B2-transfected cells; red line: control siRNA-transfected cells. The numbers in histograms are MFI ± SD. *, p<0.05; **, p<0.01; ***, p<0.001. **G,** knockdown of MS4a4B accelerated IL-2-induced proliferation of T32 cells. T32 cells were infected with either shMS4a4B- or shLuc-lentiviral vector. Infected cells (1×10^5^) were cultured in 96 well plate for 24 hr in the presence of serially diluted IL-2, followed by incubation with 1μCi 3H-thymidine for an additional 16 hr. Cell proliferation was assayed by 3H-thymidine incorporation. A representative of two independent experiments is shown. *, p<0.05; **, p<0.01.

T cell proliferation is a critical process for T cell-mediated immunity, which is reciprocally regulated at multiple levels by numerous activators and inhibitors [26], [27], [28]. Its regulation, however, is still not clearly understood. Studies on CD20 suggest that MS4A proteins may regulate cell proliferation [13], [23]. To determine the role of MS4a4B in T cell proliferation, we constructed a MS4a4B-expressing retroviral vector, which co-expresses GFP as a selection marker. We used the MS4a4B-retroviral vector to manipulate expression of MS4a4B in primary T cells, which were subsequently labeled with PHK-26, a red fluorescent dye, and analyzed T cell proliferation upon CD3/CD28 stimulation by flow cytometry. Over-expression of MS4a4B by retroviral vector indeed inhibited proliferation of primary T cells (Fig. 2A). Since primary T cells constitutively expressed high levels of MS4a4B, we next used EL4 cells (MS4a4B^−^), a widely used thymoma cell line [29], [30], [31], to confirm the inhibitory effect of MS4a4B on T cell proliferation. We constructed a lentiviral vector (LV) to express MS4a4B and a GFP marker in EL4 cells. We sorted GFP^+^ cells and generated a stable MS4a4B-expressing EL4 cell line. To test proliferation of EL4 cells, the infected EL4 cells were labeled with PHK-26 and cell proliferation was analyzed by flow cytometry. As predicted, forced expression of MS4a4B inhibited EL4 thymoma cell proliferation (Fig. 2B).

Small interfering RNA (siRNA)-mediated gene silencing has been shown to be a powerful approach for studying protein function [32]. Since forced expression of MS4a4B over physiologic levels in cells may lead to artificial data interpretation in some cases, we next proceeded to knockdown MS4a4B expression. To confirm the inhibitory role of MS4a4B in T cell propagation, we used synthesized siRNAs to silence expression of MS4a4B in T32 cells, a T helper cell line derived from normal primary T cells, which normally expresses high levels of MS4a4B protein [1]. Three siRNA duplexes were designed to target cDNA regions encoding the N-terminal region, the first extracellular domain, and the C-terminal intracellular domain of MS4a4B respectively (supplementary Fig. S3A and B). Knockdown of MS4a4B by selected MS4a4B-siRNAs markedly reduced expression of MS4a4B at both RNA transcription and protein levels (Fig. 2C, 2D and Supplementary Fig. S3C). In comparison with RNA expression, siRNA-induced reduction of MS4a4B protein was somewhat delayed, perhaps due to the longer half-life of protein. To determine the impact of MS4a4B-knockdown on cell proliferation, T32 cells pre-labeled with PHK-26 were transfected with FAM-labeled either MS4a4B-specific siRNA (siMS4a4B2) or negative control siRNA. Proliferation of the transfected cells in culture was assessed by flow cytometry. Consistent with the findings from over-expression studies, knockdown of MS4a4B accelerated proliferation of T32 cells (Fig. 2E). Similar results were observed in primary T cells from C57BL/6 mice when MS4a4B expression was knocked down by siRNA approaches (Fig. 2F), suggesting that MS4a4B plays an inhibitory role in T cell propagation. This appears not to be in line with our previous observation in which overexpression of MS4a4B enhanced IL-2 levels in activated T cells. It remains to be elucidated whether MS4a4B plays a role in survival of differentiated Th1 cells, which led to a prolonged IL-2 production in these cells. To test how MS4a4B expression impacts IL-2 responsiveness of T cells, we added serially diluted IL-2 in culture of T32 cells transduced with either shMS4a4B lentiviruses (for MS4a4B knockdown) or shLuc lentiviral vector as control and analyzed proliferation of these cells. The results showed that knockdown of MS4a4B accelerated IL-2-induced proliferation of T32 cells (Fig. 2G).

**Figure 3:**
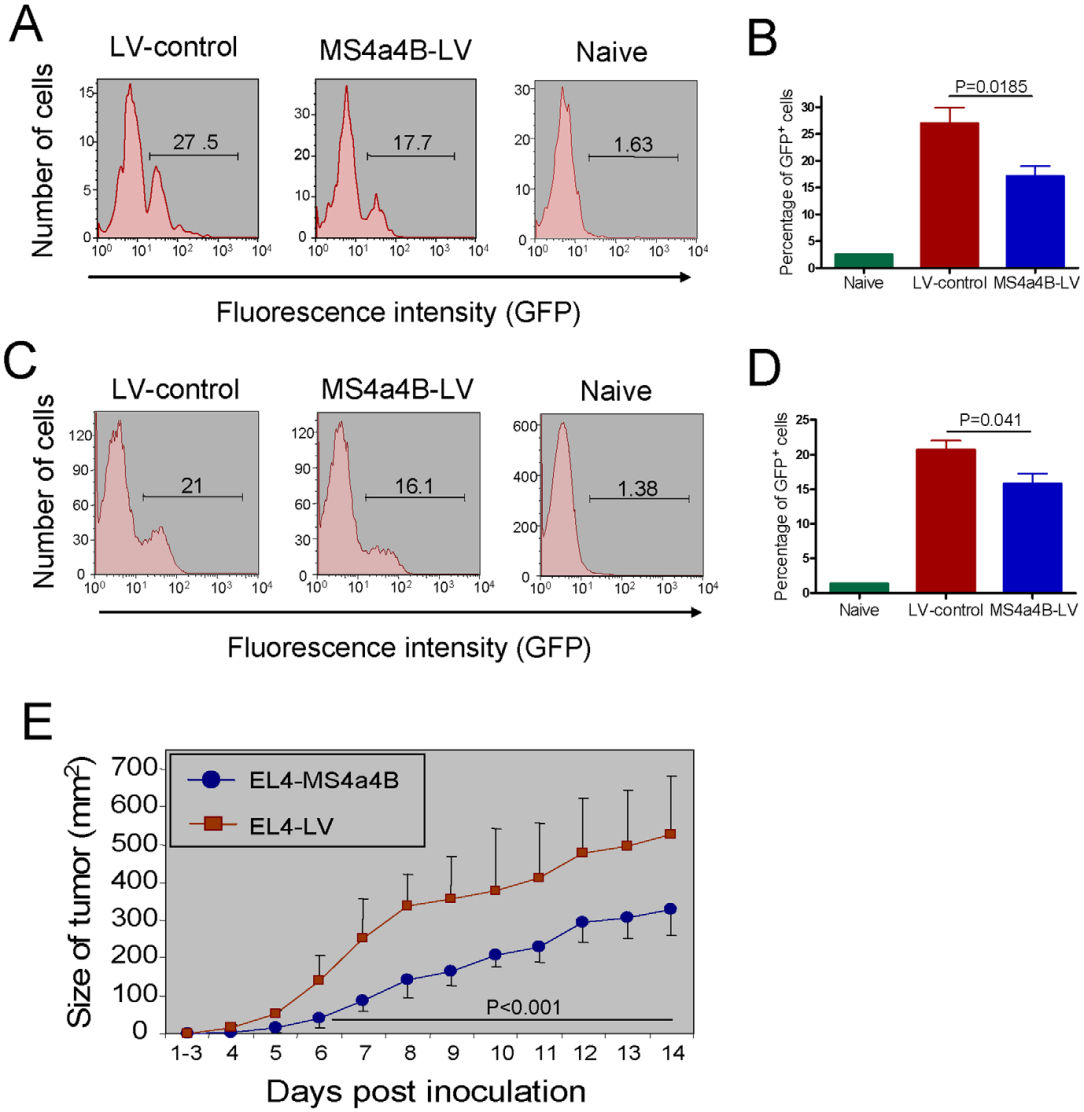
Expression of MS4a4B by lentiviral vector inhibits propagation of thymoma cells in vivo. EL4 thymoma cells were infected by MS4a4B-LV or mock LV control. The infected cells (GFP^+^) were purified by flow cytometric cell sorting (purity: >99%). 1.5×10^7^ sorted cells were infused into C57BL/6J recipients by i.v injection. Mice were sacrificed on day 3 after cell transfer. EL4 thomoma cells (GFP^+^) in blood and spleen were assessed by flow cytometry. Data shown are representative of three repeat experiments. **A,** Representative flow histograms for each group of blood samples. The number shown is percentage of GFP^+^ cells in lymphocyte gate. B, Percentage ± SD of GFP^+^ cells in blood samples from mice injected with either MS4a4B-LV-infected or mock LV-infected EL4 cells (N = 5). **C,** Representative flow histograms for each group of spleen samples. The number shown is the percentage of GFP^+^ cells in lymphocyte gate. **D,** Percentage ± SD of GFP^+^ cells in spleens from mice injected with either MS4a4B-LV-infected or mock LV-infected EL4 cells (N = 5). E, MS4a4B inhibits solid thymoma growth in vivo. 1×10^6^ EL4 cells were injected subcutaneously in the right flank of mice. Size of tumor was measured daily after inoculation. Results are presented as mean ± SD of mm^2^ (N = 8). Data shown are representative of two independent repeat experiments.

To confirm if MS4a4B does serve as a negative modulator for T cell proliferation *in vivo*, EL4 cells infected with either MS4a4B-expressing-lentivirus or mock control lentiviral vector were infused into C57BL/6 recipients. EL4 cell propagation was assessed by analyzing the percentage of infused EL4 cells in blood and spleen with flow cytometry three days after cell transfer. As indicated by the percentage of GFP-positive cells, MS4a4B-expression reduced the propagation of EL4 cells in both peripheral blood (Fig. 3A and B) and spleens (Fig. 3C and D). To exclude the possibility that MS4a4B-infected EL4 cells may dominantly migrate into organs other than spleen, we also examined brain, thymus, heart, lung, liver, kidney and intestine. The results showed that there were no infused EL4 cells (GFP^+^) detected in those organs except heart and liver, in which the level of MS4a4B-infected EL4 cells was also slightly lower than that of LV-infected EL4 cells (data not shown). To further verify the inhibitory role of MS4a4B in solid thymoma model, 1×10^6^ MS4a4B-expressing lentivirus (or control vector)-infected EL4 cells were inoculated subcutaneously in the right flank of 8–10 week-old C57BL/6 mice. Tumor growth was monitored daily after inoculation. Results showed that forced expression of MS4a4B in EL4 cells significantly reduced tumor growth in vivo (P<0.001) (Fig. 3E). This is unlikely due to host specific cellular responses to MS4a4B protein given that spleen cells from EL4-inoculated hosts did not respond to EL4 cells expressing MS4a4B when they were cocultured with EL4-MS4a4B cells in vitro (data not shown).

### MS4a4B regulates T cell proliferation by interfering cell cycle progression

**Figure 4:**
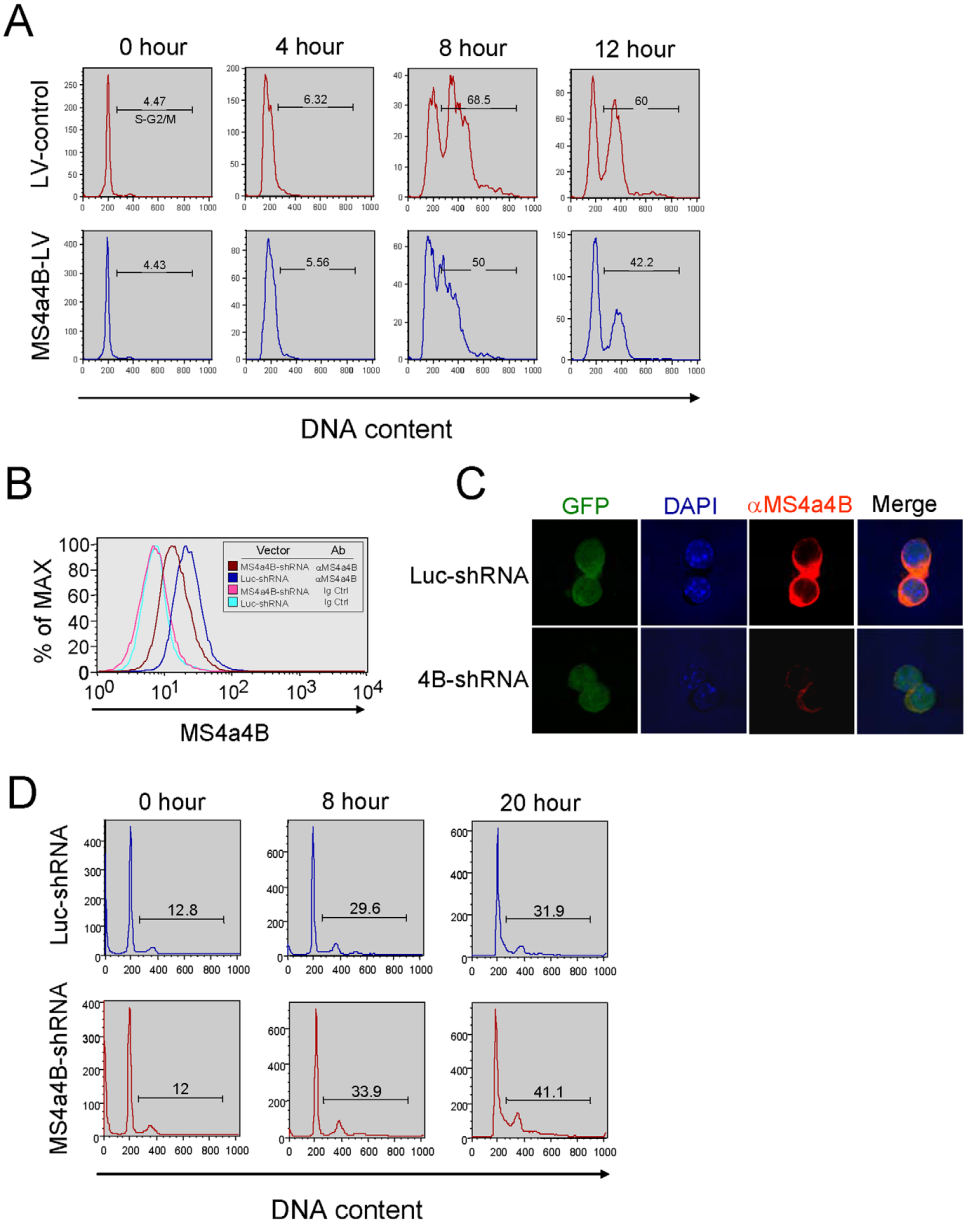
Expression of MS4a4B in T cells modulates cell cycle progression. **A,** MS4a4B-LV or mock LV-infected EL4 thymoma cells were synchronized by treatments with 2.5 mM thymidine and serum starvation. The synchronized cells were adjusted into 5 × 10^5^/ml and were stimulated with complete medium containing 15% FCS. For cell cycle analysis, cells were harvested from culture at the time indicated. DNA content in cells was determined by propidium iodide staining and flow cytometric analysis. Results are shown as histogram from each sample with numbers indicating percentage of S-G2/M phase cells. Data presented are representative of three repeat experiments. **B,** Infection of T32 cells by shMS4a4B-expressing LV vector decreased MS4a4B protein expression. T32 cells stably infected with shMS4a4B-LV or shLuc-LV control were sorted by flow cytometry. MS4a4B expression in the sorted T32 cells was assessed by flow cytometry with anti-MS4a4B staining. Data are shown as representative histograms from analysis with Flowjo. **C,** shMS4a4B-LV or shLuc-LV control-infected T32 were co-stained with DAPI and anti-MS4a4B antibody, followed by labeling with anti-rabbit IgG-Cy3 conjugate. Knockdown of MS4a4B protein in T32 cells by shMS4a4B-LV was examined by confocal microscopy (Magnification, ×40). **D,** Knockdown of MS4a4B expression in T32 cells by shMS4a4B-LV inhibited cell cycle progression. shMS4a4B-LV or shLuc-LV control-infected T32 cells were synchronized as described in “A”. Synchronized cells were stimulated by 15% FCS RPMI medium containing 20 U/ml IL-2. Cell samples were harvested from culture at the time indicated. Cell cycle was analyzed by propidium iodide staining. Data are shown as the representative histogram of three repeat experiments. The numbers in histogram are the percentage of cells in S-G2/M phase.

It has been documented that CD20 regulates B cell proliferation by impacting cell cycle progression in these cells [22], [33]. HTm4 was found to prevent cell cycle progression from G0/G1 phase into S-G2/M phase in hematopoeotic cells [15], [24]. To get some insight into how MS4a4B modulates T cell proliferation, we tested if MS4a4B protein regulates cell cycle progression by using MS4a4B-lentivirus-infected EL4 cells. EL4 cells stably infected by MS4a4B-lentivirus (or mock lentiviral vector as control) were synchronized by two cycle treatment with 2.5 mM thymidine and serum starvation. Synchronized cells then were stimulated with complete medium containing 15% FCS. Cell samples were collected at serial time points after stimulation and cell cycle was analyzed by flow cytometry with propidium iodide staining. After 8 hours of stimulation, 68.5% of control cells entered S-G2/M phase. In contrast, the percentage of cells that entered S-G2/M phase was markedly reduced in MS4a4B-expressing vector-infected EL4 cells (Fig. 4A). These results suggest that MS4a4B protein inhibits cell proliferation by preventing cell cycle progression from G0/G1 phase into S-G2/M phases.

To further confirm the role of MS4a4B in cell cycle regulation by knockdown approaches, we constructed a lentiviral vector expressing short hairpin RNA (shRNA) for MS4a4B (MS4a4B-shRNA), which was designed based on the targeting sequence (siMS4a4B2) selected by siRNA knockdown experiments described above. Knockdown by MS4a4B-shRNA vector markedly reduced MS4a4B mRNA (supplementary Fig. S4) and protein expression (Fig. 4B and C). To determine the impact of MS4a4B-knockdown on cell cycle, shRNA-targeting vector-infected T32 cells were isolated by flow cytometric cell sorting to achieve >99% of GFP expression. The purified T32 cells were synchronized as described for EL4 cells above. The cells then were stimulated by complete medium containing 15% FCS. Cell samples from culture were analyzed for cell cycle by propidium iodide staining and flow cytometric analysis. Consistent with the observation from over-expression experiments, knockdown of MS4a4B protein promoted cell cycle progression from G0/G1 phase into S-G2/M phase (Fig. 4D), indicating that reduction of MS4a4B protein released cells from MS4a4B-mediated suppression of cell cycle.

### MS4a4B-mediated cell cycle inhibition is correlated with enhanced expression of cell cycle inhibitors and reduced Cdk2 activity

Cell cycle progression is driven by the temporal induction and activation of cyclin-dependent kinases (CDKs) [34]. Activation of CDKs requires the formation of CDK-cyclin complexes and is negatively regulated by two groups of CDK inhibitory proteins: Ink4 family (p16, p15, p18 and p19) and Cip/Kip family (p21, p27 and p57) [35]. Binding of Ink4 inhibitors to CDK4 or CDK6 prevents CDK interaction with cyclin D in G1 phase, which interferes with cell cycle progression from G0 to S phase. CDK inhibitors of the Cip/Kip family more broadly inhibit activity of cyclin-CDK complexes involving CDK2 and cyclin D, E and A, which causes G1 arrest and suppression of cell cycle progression beyond G1 phase.

**Figure 5:**
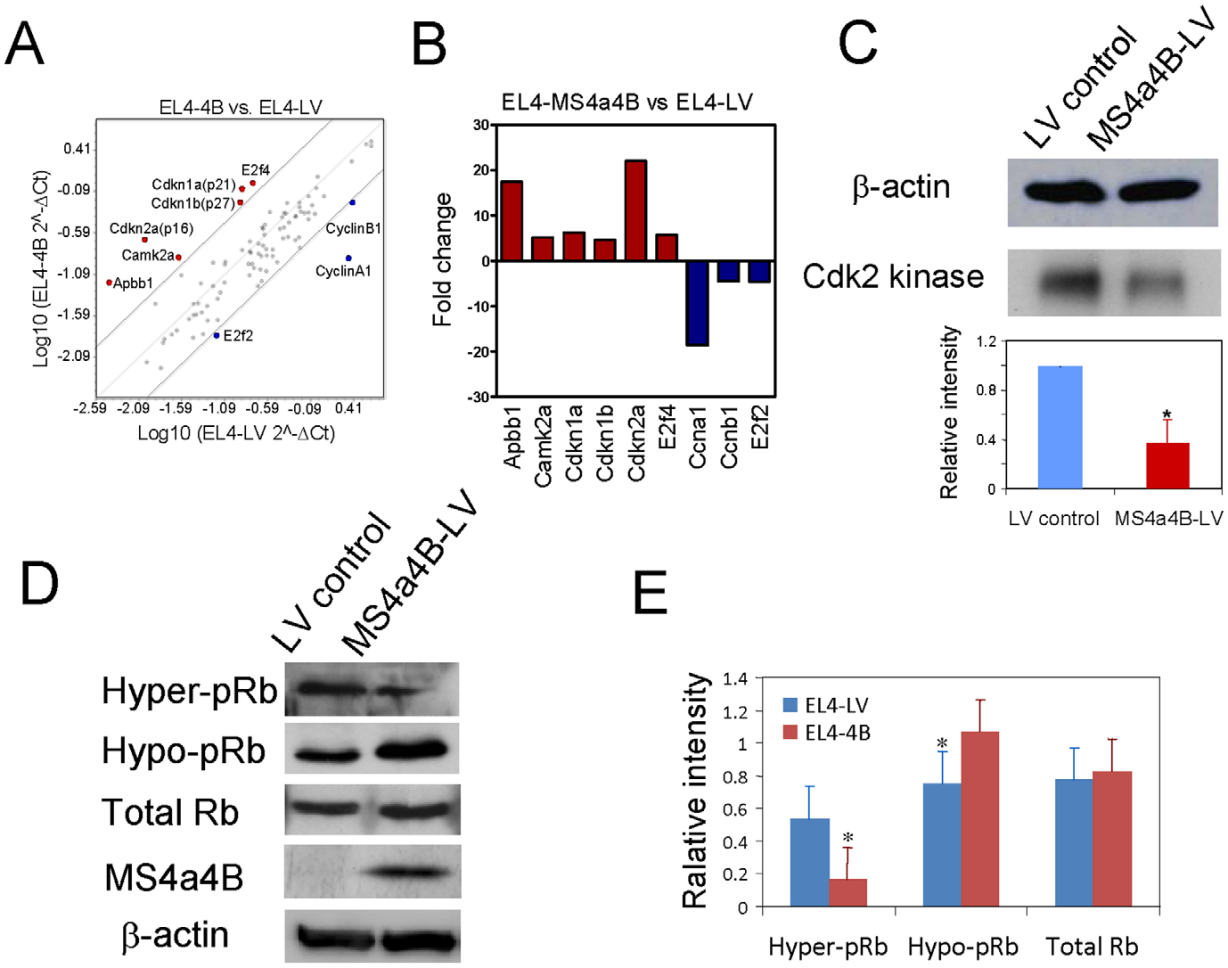
Expression of cell cycle-regulatory genes in MS4a4B-LV-infected EL4 cells. **A,** Total RNA was isolated from MS4a4B-LV vector- or mock LV vector-infected EL4 cells. Expression of 84 key regulatory genes on cell cycle progression was determined with real time PCR array analysis kit (Cat^#^: PAMM-020, SABiosciences, Gaithersburg, MD). Data were analyzed with on-line analysis software according to the manufacturer's instructions and presented as scatter plot with fold change of genes. Red dot: genes increased by ≥4 fold; blue dot: genes decreased by ≥4 fold. **B,** Genes with fold change ≥4 were shown as column figure. **C,** MS4a4B (or control) vector-infected EL4 cells were synchronized and stimulated with 15% FCS for 8 hours. Cell lysates (100 μg) were immunoprecipitated with anti-Cdk2-protein A beads. Cdk2-cyclin activity was assayed as described in “Methods”. The same samples (25 μg) were used for western blot with anti-β-actin antibody to ensure identical loading. Relative intensity (sample vs. LV control) is shown in the lower panel. *, p<0.05. **D,** Cell lysates described in “C” were separated on 10% SDS gel followed by western blotting with appropriate antibodies as indicated. E. Relative intensity of blots in “D” (sample vs. internal control (β-actin)) was determined by densitometry. *, p<0.05.

To dissect the underlying mechanism of MS4a4B-mediated regulation of cell cycle, we analyzed gene expression that regulates cell cycle transition by real-time PCR array. We found that expression of several inhibitory genes was enhanced in MS4a4B-lentivirus-infected EL4 cells, including Ink4 family protein (p16), Cip/Kip family proteins (p21 and p27) and E2f4, the latter having been found to repress gene transcription by interfering with the binding of E2f1, E2f2 and E2f3 to promoters [36]. On the other hand, expression of positive cell cycle regulators, e.g. Cyclin A1, Cyclin B1 and E2f2, was decreased (Fig. 5A and B). Another enhanced inhibitory molecule is Apbb1 (Amyloid beta (A4) precursor protein-binding, family B, member 1), also named Fe65, which is an adaptor protein localized in the nucleus. Overexpression of Fe65 in mouse fibroblasts has been shown to block cell growth by inhibiting the activation of a key S phase gene, the thymidylate synthase (TS) gene [37]. Notably, MS4a4B expression enhanced the production of the calcium/calmodulin-dependent protein kinase (CaM kinase) IIα, also known as Camk2a, whose kinase activity is dependent upon its activation by calmodulin (CaM). CaM is one of the key proteins that transduces a signal in response to increases in intracellular Ca^2+^. Whether MS4a4B protein is involved in regulation of Ca^2+^ signaling still remains to be determined. We observed no change in p53 at RNA transcription levels, suggesting that p53 is not involved in MS4a4B-mediated inhibition of cell cycle.

Since overexpression of MS4a4B was correlated with reduced levels of cyclin A1, which has been documented to interact with Cdk2, we next examined Cdk2 kinase activity in MS4a4B-lentivirus-infected EL4 cells by Ckd2 kinase assay. We found that overexpression of MS4a4B markedly attenuated Cdk2 kinase activity (Fig. 5C). We further examined whether inhibition of Cdk2 activity subsequently resulted in higher levels of hypophosphorylated retinoblastoma protein (Rb), a crucial suppressor protein for gene transcription, and cell cycle progression. Indeed, levels of hopophosphorylated Rb were markedly elevated, which was accompanied by decreased levels of hyperphosphorylated Rb, in MS4a4B-lentivirus-infected EL4 cells in comparison with MS4a4B^−^-EL4 cells (Fig. 5D and 5E).

## Discussion

It has been shown previously that MS4a4B is selectively expressed in Th1 cells [1], [20] and regulatory T cells (Treg) [19]. In primary T cells, MS4a4B is associated with Th1-cytokine production and its expression is repressed by Stat6-signaling [20]. In Treg cells, interaction of MS4a4B with GITR augments GITR signaling and T cell IL-2 production [19]. In this study, we demonstrated, for the first time, that MS4a4B plays an inhibitory role in T cell proliferation. Although MS4a4B is highly expressed in mature primary T cells, it is absent in malignant T cells. Interestingly, MS4a4B expression is upregulated during activation of primary T cells and overexpression of MS4a4B by viral vectors downregulates T cell proliferation, suggesting that MS4a4B may serve as “survival modulator” to protect activated cells from overgrowth. On the other hand, silence of MS4a4B in thymoma and T hybridoma cells may partially explain the uncontrollable growth of malignant T cells. Absence of MS4a4B expression in thymoma cells is likely due to active suppression of MS4a4B gene transcription by unknown factors derived from tumor cells rather than mutation of the MS4a4B gene itself since T hybridoma cells also lose MS4a4B expression (Table 1), in which MS4a4B gene should be compensated by the alleles from normal T cells. Of note, MS4a4B is also expressed at low levels in bone marrow cells. It is unclear whether this proportion of bone marrow cells represents the hematopoietic progenitors that have the potential to migrate into the thymus and are committed to T cell lineage.

CD20 and HTm4 have been shown to regulate proliferation of activated B cells and hematopoietic cells respectively by interfering with cell cycle progression [15], [22]. Although the mechanisms underlying CD20 or HTm4-mediated regulations are not fully understood, available evidences support that they are using different mechanisms. CD20 is thought to be a regulator of transmembrane Ca^2+^ conductance which indirectly impacts cell cycle progression and proliferation of B cells [11], [38]. Antibody binding to a CD20 epitope (B1) hence inhibits cell cycle progression of B cells [22]. HTm4 has been found to bind to cyclin-dependent kinase-associated (CDK-associated) phosphatase-CDK2 (KAP-CDK2) complexes by its C-terminal region and to stimulate the phosphatase activity of KAP, which subsequently causes cell cycle arrest at the G0/G1 phase. Cell membrane localization of MS4a4B protein in T cells is similar to that of CD20 in B cells but differs from that of HTm4, which is mainly localized on nuclear membrane [15]. It remains to be determined whether MS4a4B can serve as a calcium channel, or as a regulatory protein for cell receptor (as CD20 does in B cells [11], [39]). In comparison with CD20, both MS4a4B and CD20 regulate cell cycle and cell proliferation. However, the effect of MS4a4B on cell cycle and cell proliferation in T cells seems different from that of CD20 in B cells since expression of recombinant CD20 was shown to accelerate cell cycle progression [21]. This difference may explain why B lymphoma cells conserve expression of CD20 while tumor T cells eliminate MS4a4B expression. In contrast, the inhibitory role of MS4a4B on cell cycle and cell proliferation is similar to that of HTm4 in hematopoietic cells, in which overexpression of HTm4 causes cell cycle arrest at the G0/G1 phase [15]. These data suggest that function of MS4A proteins may vary in different type of cells despite the homology in amino acid sequences and the similarity in structure among the MS4A gene family.

Data from our studies thus far show that MS4a4B inhibits cell cycle progression of T cells possibly by modulating levels of cell cycle regulatory elements. In primary T cells, CD4^+^ T cell line and thymoma cells, as confirmed by both overexpression and knockdown approaches, MS4a4B is correlated with increase of cell cycle inhibitors, e.g., p16^Ink4a^, p21^Cip1^ and p27^Kip1^, and decrease of cyclin A and B. Furthermore, overexpression of MS4a4B caused reduction of Cdk2 activity and promoted dephosphorylation of Rb. Cdk2 is a crucial molecule that drives cell cycle transition from G0/G1 to S-G2/M phases [40]. Downregulation of Cdk2 activity will therefore result in dephosphorylation of Rb, subsequently leading to inhibition of gene transcription and cell cycle arrest. To date, we have no data to show whether MS4a4B inhibits Cdk2 activity by interacting with other regulatory molecules, e.g., KAP, as HTm4 does. However, evidences from our studies support the hypothesis that MS4a4B more likely causes reduction of Cdk2 activity by impacting upstream of the signaling pathway since overexpression of MS4a4B in EL4 cells also increases Camk2a production. It has been documented that activation of Camk2a may regulate cell cycle in either a negative or positive manner under different circumstances [41], [42]. In case of MS4a4B expression in EL4 cells, MS4a4B protein may facilitate the Ca^2+^/CaM signaling pathway by impacting intracellular Ca^2+^ activity and cause upregulation of Camk2a, which promotes the transcription of Ink4 and Cip/Kip family Cdk inhibitors. Although machinery interplays in these processes still remain to be elucidated in detail, our data suggest that MS4a4B may modulate cell cycle transition by upregulation of cell cycle inhibitors and down-regulation of cell cycle activators possibly through the Ca^2+^/CaM signaling pathway.

Cell proliferation and differentiation are two closely related but independent processes [43]. T cell activation and proliferation are essential for T cell differentiation [44]. Proliferating T cells usually have several fates under certain circumstances: toward terminal differentiation, or exit from cell cycle for resting and survival, or undergo apoptosis. It has been documented that TcR-stimulatory signals trigger T cell proliferation but preactivated T cells may undergo activation-induced cell death (AICD) in response to the same signals [45]. In our study, we found that MS4a4B expression was upregulated in activated T cells and decrease of MS4a4B was associated with apoptosis of T cells (Figure 1C and 1E). Given that MS4a4B can inhibit T cell proliferation, this raises the possibility that in addition to its anti-proliferative role, MS4a4B expression may prolong survival of activated T cells by preventing T cells from apoptosis. Currently, we have no evidence to show whether down-regulation of MS4a4B in apoptotic T cells is a cause or consequence of T cell apoptosis. If MS4a4B does have an anti-apoptotic effect and provide survival signals for activated T cells, that may explain why overexpression of MS4a4B in T cells enhanced IL-2 levels during T cell activation as we observed in our previous studies [1].

In conclusion, our study provides evidence that MS4a4B modulates T cell proliferation as a negative regulator by inhibiting cell cycle transition from G0/G1 phase into S-G2/M phase. Given that MS4a4B expression in primary T cells is upregulated after T cell activation, MS4a4B likely contributes to negative feed-back regulatory pathways, which will self-limit over-propagation of activated T cells. In other words, it may facilitate activated T cells for long-term survival. This may account for its high levels of expression in mature T cells conserved by evolution. On the other hand, it may partially explain the uncontrollable growth of malignant T cells, e.g. thymoma. Since mature T cells express high levels of MS4a4B while malignant T cells lose its expression, MS4a4B may also serve as a biomarker to distinguish normal mature T cells from tumor T cells. Considering the structural and functional similarity between MS4a4B and CD20, as well as HTm4, this regulatory role of MS4a4B and other members of the MS4A gene family in cell proliferation could be fundamental for cellular biology in general. In addition, taking into count the importance of CD20 as a target of antibody-based immune therapeutics for B cell-mediated diseases, MS4a4B may represent a potential target on T cells for similar antibody-based therapeutics of T cell-mediated immune diseases.

## Materials and Methods

### Ethics Statement

All experiments were performed in accordance with National Institutes of Health and Thomas Jefferson University guidelines. The study involving vertebrate animals was approved by the Thomas Jefferson University Institutional Animal Care and Use Committee (IACUC, Protocol #833A).

### Mice and cells

C57BL/6J mice, 8 to 10 weeks old, male (The Jackson Laboratory), were used for *in vivo* study and as donors for primary cells. EL4 and HEK 293T cells were from American Type Culture Collection (Manassas, VA). T cell clone T32 was obtained from D. Scott (University of Maryland School of Medicine). All cells were cultured with RPMI 1640 complete medium containing 10% of fetal calf serum (FCS) except for HEK 293T cells, which were cultured with DMEM complete medium containing 10% FCS.

### Antibodies and immune staining

Antibodies used for immunostaining mouse cells were obtained from BD-Biosciences (San Diego, CA). Antibodies against C-terminal of MS4a4B were generated as described previously [1] and were biotinylated with SureLINK Chromophoric Biotin Labeling Kit (KPL, Gaithersburg, MD). MS4a4B intracellular staining was performed as described previously [1]. Flow cytometry was performed using a FACSCalibur (BD-Biosciences). Data were analyzed with FlowJo software (Tree Star, Ashland, OR).

### Western blot analysis

Cells were lysed in lysis buffer (Cell Signaling) supplemented with protease inhibitor (Complete Mini, EDTA-free; Roche Applied Science). Cell lysates were separated by 10% SDS-PAGE and transferred onto Immun-Blot PVDF membrane (Bio-Rad Laboratories). Membranes were blotted with primary antibodies followed by incubation with HRP-conjugated secondary antibodies. The blots were developed by ECL reagents and exposed on HyperFilm™ (Amersham). The following antibodies were used for western blotting: Cdk2 (H-298) and β-Actin (AC-15) (Santa Cruz Biotechnology); Rb (N-terminal) (BioLegend); Rb (Ab-807, Signalway Antibodies); Phospho-Rb (Ser807/811) (Cell Signaling) and anti-MS4a4B (C-terminal) antibody [1].

### Immunohistological staining and confocal microscopy

T cells were seeded on poly-L-lysine–coated chamber slides in culture medium. For immune staining, cells were fixed with 4% paraformaldehyde plus 0.5% glutaraldehyde and then were permeabilized with 0.5 ml of 0.2% Triton X-100. After blocking with 10% horse serum, slides were incubated with primary antibody at 4°C overnight, followed by staining with Cy3-labeled anti-rabbit IgG conjugate (Jackson ImmunoResearch Lab). The stained slides were covered with mounting medium (Vector Laboratories). Results were visualized by confocal microscopy (Zeiss LSM 510).

### Viral vectors for MS4a4B-overexpression

MS4a4B-GFP-MIGR retroviral vector was constructed as described previously [1]. MS4a4B-lentivirus vector was prepared by inserting MS4a4B-encoding sequence into a bicistronic lentiviral vector containing GFP marker [46]. The lentiviral vector particles were produced in HEK 293T packaging cells by using the three-plasmid transient transfection system [47].

### Transfection and MS4a4B knockdown by siRNA

Three siRNA candidates were selected by using online siRNA design software to target different regions of the MS4a4B gene (supplementary Fig. S3). The synthesized siRNA duplexes were labeled with FAM at 5′-end of sense oligonucleotide of the duplex. FAM-labeled Silencer Negative Control #1 siRNA was purchased from Ambion, Applied Biosystems (Cat^#^:AM4611). Cell transfection was performed using Lipofectamine 2000 per the manufacturer's protocol (Invitrogen). Knockdown efficiency was assessed by flow cytometry with anti-MS4a4B antibodies and by RT-PCR with MS4a4B-specific primers [1].

### Construction of MS4a4B-shRNA and Luc-shRNA-lentiviral vectors

Based on the knockdown efficiency of synthesized siMS4a4B duplexes, we selected encoding sequence 630–649 of MS4a4B (NCBI GenBank NM_021718) as target and shRNA for luciferase (Luc) as irrelevant control. MS4a4B-shRNA and Luc-shRNA fragments were synthesized by Sigma. shRNA-knockdown lentiviral vectors were constructed with a Gateway-based cloning system (Invitrogen). Briefly, selected shRNA fragments were inserted into pEN-mH1c entry vector (ATCC ID10326369) at AflII/XhoI sites. Pol III-shRNA cassette from pEN-mH1c vector was in turn subcloned into pDSL-hpUGIH plasmid by LR recombination reaction to generate shRNA expression lentiviral vectors (supplementary Fig. S4). For virus packaging, HEK 293T cells were transfected with pDSL-hpUGIH plasmid containing shRNA expression cassette by using the three-plasmid transient transfection system [47].

### Viral infection of T cells

Primary T cell infection was performed as described previously [1]. Viral infection of T cell line and thymoma cells was performed as described for primary T cells except that there was no prestimulation.

### Cell labeling and proliferation assay

T cell lines or primary T cells primed with either MS4a4B over-expression approach or MS4a4B-knockdown approach were labeled with PHK-26 red fluorescent dye (Sigma) according to the manufacturer's instruction. Cell proliferation was measured by flow cytometry. To assess proliferation of MS4a4B-virus or control vector-infected EL4 cells *in vivo*, 1.5×107 cells were infused into C57BL/6J recipient mice. Blood and spleen samples were harvested from mice on day 3 after cell transfer. Percentage of EL4 cells was determined by flow cytometry according to GFP expression.

### Cell cycle analysis

EL4 cells stably infected by either MS4a4B-expressing lentiviruses or mock vector were isolated by flow cytometric cell sorting (GFP expression >99%). Cells were then synchronized by two cycle treatments with 2.5 mM thymidine for 20 h (in complete medium containing 10% FCS for the first treatment and 0.5% FCS for the second treatment) with a 10-hour interval. Cells were collected from culture at serial time points. Cell cycle was analyzed by propidium iodide staining [48].

### Cdk2 kinase assay

Cell lysates (100 μg) were immunoprecipitated with anti-CDK2-protein A beads. Immunoprecipitated protein was incubated at 30°C for 30 min with 2 µCi γ-P32-ATP and Histone H1 (Roche Diagnostics) as a substrate in kinase buffer (50 mM Hepes pH7.0, 10 mM MgCl2, 10 mM DTT) containing 30 μM ATP. The reaction was stopped by adding 2X sample buffer and boiled for 3 min before separation on a 12% SDS-PAGE. The gel was dried and exposed to X-ray film.

### Statistical analysis

Statistical analysis was performed using a two-tailed t-test except EL4 solid tumor model, for which two-way ANOVA test was used to determine statistical difference between groups. A value of p<0.05 is considered statistically significant.

## Supporting Information

Figure S1 Expression of MS4a4B in NK cells, macrophages and bone marrow cells. A, Spleen cells from C57BL/6J mice were surface-stained with anti-Mac1-FITC and anti-NK1.1-PE, followed by intracellular staining with biotinylated-anti-MS4a4B antibody (blue line) or biotinylated-Ig control (red line), which were subsequently labeled by streptavidin-Red 670 conjugate. For flow cytometric analysis, cells were first gated by Mac1 and NK1.1. Mac1+NK1.1- cells (macrophage-enriched population) and Mac1+NK1.1+ cells (Mac1+ NK cells) were then analyzed respectively for MS4a4B expression. The representative of three repeat experiments is shown. B, Bone marrow cells from C57BL/6J mice were surface-stained with anti-Mac1-FITC, followed by intracellular staining with anti-MS4a4B antibody (blue line) or Ig control (red line) as described in “A”. For flow cytometric analysis, cells were first gated by Mac1. Mac1+ and Mac1- cells were then analyzed respectively for MS4a4B expression. The representative of three repeat experiments is shown.
(0.66 MB TIF)

Figure S2 MS4a4B expression is absent in malignant T cells. Thymoma cells (A), T hybridoma cells (B) and T32 cell line (C), as positive control) were stained by intracellular staining with biotinylated-rabbit anti-MS4a4B antibody (or biotinylated-rabbit IgG as control), followed by labeling with Streptavidin-PerCP-Cy5.5 conjugate. Data are presented as dot plot with percentage of MS4a4B+ cells. On representative of three independent experiments is shown.
(0.82 MB TIF)

Figure S3 Targeting MS4a4B by synthesized siRNA duplexes. A, Targeting location in MS4a4B encoding cDNA (NCBI GenBank NM_021718). B, Sequences of FAM-labeled siMS4a4Bs. C, MS4a4B expression in siRNA-transfected T32 cells. T32 cells were transfected with siMS4a4B or negative control siRNA. Cells were harvested from culture on day 4 after transfection. MS4a4B expression in transfected cells was determined by flow cytometry with anti-MS4a4B antibody. Red line: negative control siRNA-transfected cells (MS4a4B:75.4%); blue line: siMS4a4B-transfected cells.
(0.59 MB TIF)

Figure S4 Construction of shRNA-expressing lentiviral vectors. A, Structure of targeting lentiviral vector. B, Predicted shRNA transcripts. C, Knockdown of MS4a4B expression by shMS4a4B2 lentiviral vector. MS4a4B-RNA expression in either shMS4a4B- or shLuc-lentivirus-infected T32 cells was determined by RT-PCR with MS4a4B-specific primers or HPRT primers as internal control. PCR products were separated on 1% agarose gel. D, Bands in “C” were analyzed by densitometry. Results are presented as density of each sample with percentage of knockdown on columns.
(0.58 MB TIF)
