# Supplementary figures and images for: MS4a4B, a CD20 Homologue in T Cells, Inhibits T Cell Propagation by Modulation of Cell Cycle
Source: PLoS One. 2010 Nov 1;5(11):e13780. doi: 10.1371/journal.pone.0013780 (PMC2967469; doi:10.1371/journal.pone.0013780)

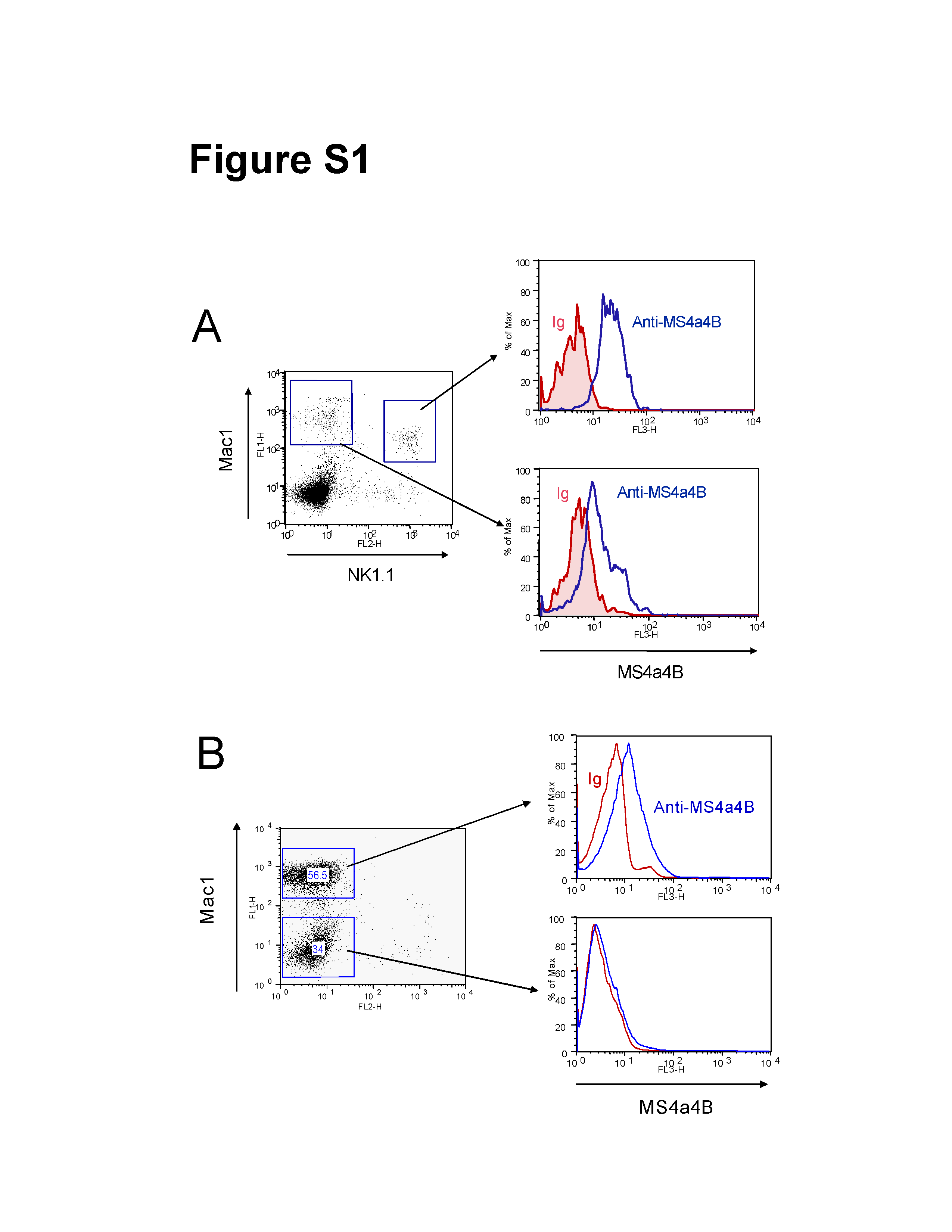

Supplement: Figure S1 — Expression of MS4a4B in NK cells, macrophages and bone marrow cells. A, Spleen cells from C57BL/6J mice were surface-stained with anti-Mac1-FITC and anti-NK1.1-PE, followed by intracellular staining with biotinylated-anti-MS4a4B antibody (blue line) or biotinylated-Ig control (red line), which were subsequently labeled by streptavidin-Red 670 conjugate. For flow cytometric analysis, cells were first gated by Mac1 and NK1.1. Mac1+NK1.1- cells (macrophage-enriched population) and Mac1+NK1.1+ cells (Mac1+ NK cells) were then analyzed respectively for MS4a4B expression. The representative of three repeat experiments is shown. B, Bone marrow cells from C57BL/6J mice were surface-stained with anti-Mac1-FITC, followed by intracellular staining with anti-MS4a4B antibody (blue line) or Ig control (red line) as described in “A”. For flow cytometric analysis, cells were first gated by Mac1. Mac1+ and Mac1- cells were then analyzed respectively for MS4a4B expression. The representative of three repeat experiments is shown. (0.66 MB TIF) [file pone.0013780.s001.tif]

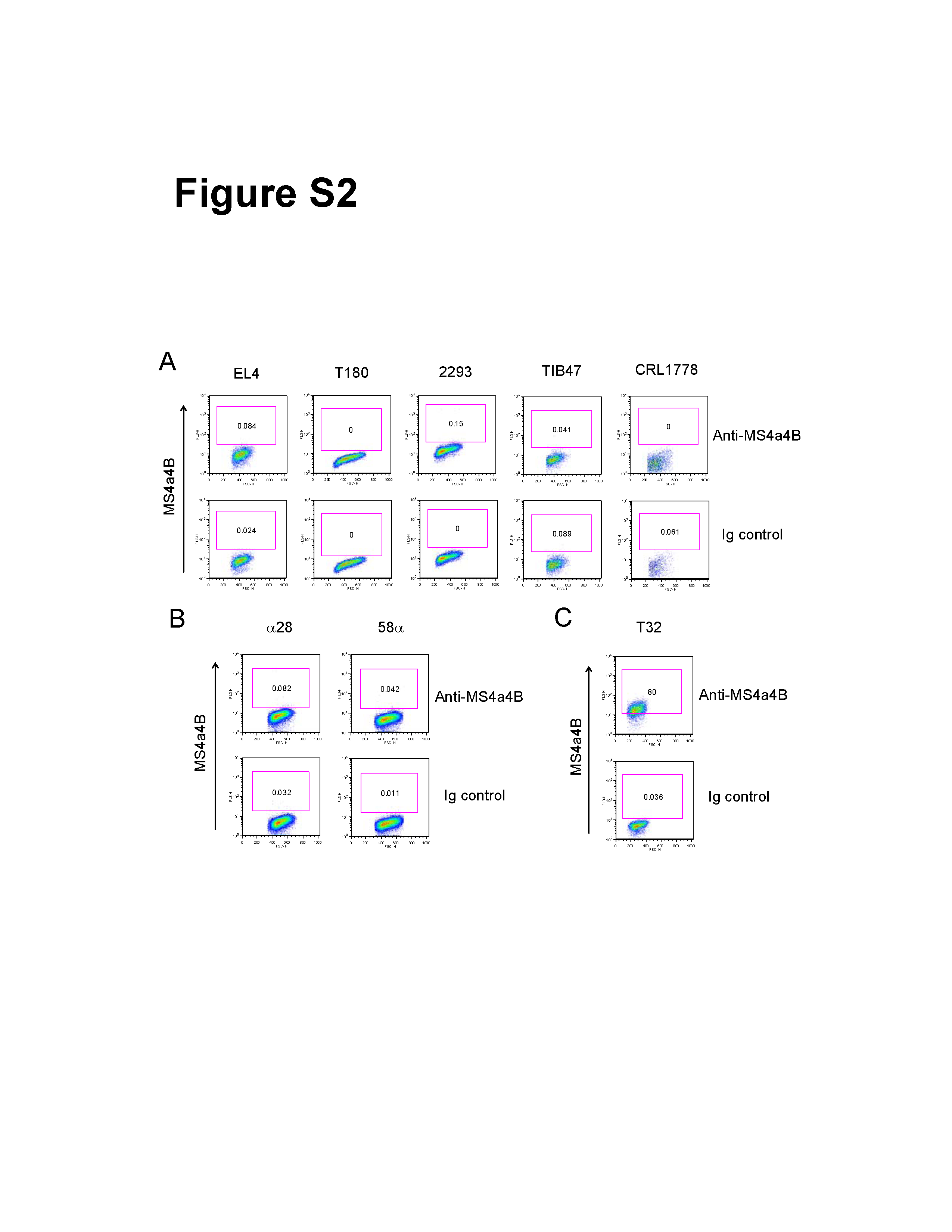

Supplement: Figure S2 — MS4a4B expression is absent in malignant T cells. Thymoma cells (A), T hybridoma cells (B) and T32 cell line (C), as positive control) were stained by intracellular staining with biotinylated-rabbit anti-MS4a4B antibody (or biotinylated-rabbit IgG as control), followed by labeling with Streptavidin-PerCP-Cy5.5 conjugate. Data are presented as dot plot with percentage of MS4a4B+ cells. On representative of three independent experiments is shown. (0.82 MB TIF) [file pone.0013780.s002.tif]

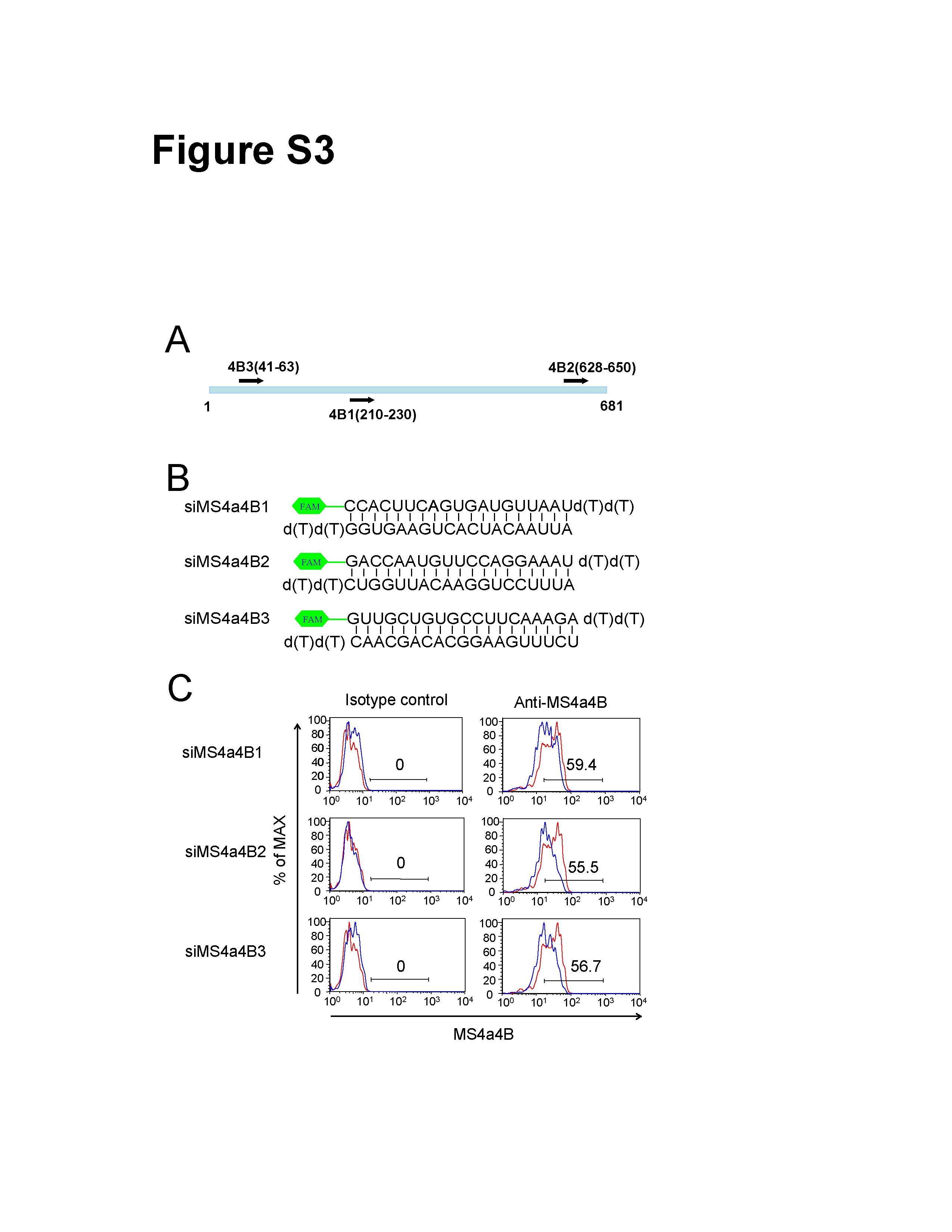

Supplement: Figure S3 — Targeting MS4a4B by synthesized siRNA duplexes. A, Targeting location in MS4a4B encoding cDNA (NCBI GenBank NM_021718). B, Sequences of FAM-labeled siMS4a4Bs. C, MS4a4B expression in siRNA-transfected T32 cells. T32 cells were transfected with siMS4a4B or negative control siRNA. Cells were harvested from culture on day 4 after transfection. MS4a4B expression in transfected cells was determined by flow cytometry with anti-MS4a4B antibody. Red line: negative control siRNA-transfected cells (MS4a4B:75.4%); blue line: siMS4a4B-transfected cells. (0.59 MB TIF) [file pone.0013780.s003.tif]

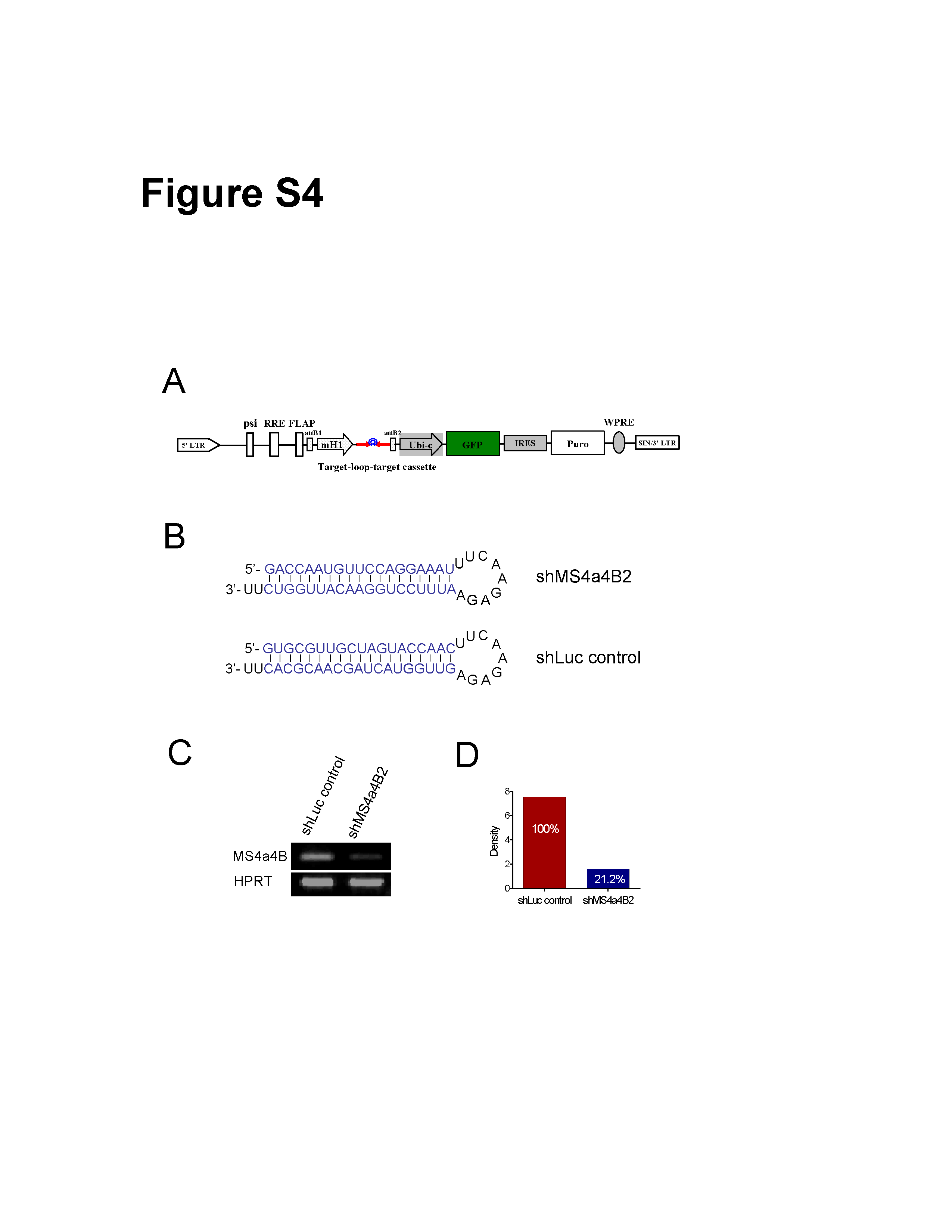

Supplement: Figure S4 — Construction of shRNA-expressing lentiviral vectors. A, Structure of targeting lentiviral vector. B, Predicted shRNA transcripts. C, Knockdown of MS4a4B expression by shMS4a4B2 lentiviral vector. MS4a4B-RNA expression in either shMS4a4B- or shLuc-lentivirus-infected T32 cells was determined by RT-PCR with MS4a4B-specific primers or HPRT primers as internal control. PCR products were separated on 1% agarose gel. D, Bands in “C” were analyzed by densitometry. Results are presented as density of each sample with percentage of knockdown on columns. (0.58 MB TIF) [file pone.0013780.s004.tif]
